# The impact of the Sri Lankan economic crisis on medication adherence: An online cross-sectional survey

**DOI:** 10.1016/j.dialog.2023.100137

**Published:** 2023-05-18

**Authors:** Ranil Jayawardena, Wasana Kodithuwakku, Piumika Sooriyaarachchi

**Affiliations:** a Department of Physiology, Faculty of Medicine, University of Colombo, Sri Lanka; b Health and Wellness Unit, Faculty of Medicine, University of Colombo, Sri Lanka; c School of Exercise & Nutrition Sciences, Faculty of Health, Queensland University of Technology (QUT), Australia

**Keywords:** Economic crisis, Medication adherence, Chronic diseases, Sri Lanka

## Abstract

**Background:**

The economic crisis in Sri Lanka has disarrayed the country's healthcare services, posing a challenge to people with chronic diseases on routine care. This study investigated the changes in medication adherence during the economic crisis.

**Methods:**

A web-based cross-sectional survey was undertaken in July–August 2022. It assessed socio-demographics, diseases, medication adherence, and reasons for changes in compliance of respondents and their family members during the economic crisis. Descriptive statistics and multivariable logistic regression analysis were used.

**Findings:**

A total of 1214 respondents, aged ≥18 years were included in the survey. The majority were females (60%). The main finding was that 39%, 41%, and 37% of participants, their family members, or children respectively have changed medication use during the crisis. Among those who changed their medication practices, the most significant change was the change in the brand, reported by 44.7% of the respondents. A similar pattern was observed among other family members, with 61.3% of adults and 53.8% of children switching brands. Respondents who lived outside the Colombo district had a significantly increased risk of changing medication (OR = 1.425, 95% CI = 1.020–1.992, *P* = 0.038). Respondents with monthly incomes of less than 100,000 LKR had a twofold greater risk of medication nonadherence compared to participants who earned more than 100,000 LKR per month (OR = 2.278, 95% CI = 1.37–3.78, *P* = 0.001). The most stated reason for changing medication among adults was the high cost of drugs, whereas among, children, the lack of access to drugs in the public or private sector was the leading cause of non-compliance.

**Interpretation:**

The population's adherence to medication is negatively impacted by the economic crisis in Sri Lanka.

## Highlights

• The economic downfall has negatively affected the medication intake in Sri Lankans.
• People outside Colombo, earning low monthly salaries showed the highest nonadherence.
• The increased cost of medications was the biggest obstacle to medication adherence.
• To identify the vulnerable groups, more thorough national-level surveys are needed.

## 1 Introduction

Chronic diseases can be defined as those which last a year or more and necessitate continuous medical attention while limiting activities of daily living [1]. Chronic disease continue to pose a huge burden on healthcare despite scientific advances. It has been identified that three out of five deaths worldwide can be attributed to chronic diseases such as cardiovascular disease, diabetes, cancer, and chronic lung disease [2]. Over one-third of the global adult population lives with two or more chronic conditions, which underlies a disproportionate amount of health expenses [3]. The significant burden of chronic diseases results in deleterious effects on national and global economies, while adverse economic conditions invariably hinder the successful management of those chronic diseases in return.

More than half of the global burden from coronary artery disease (CAD) is estimated to befall people of the Indian subcontinent in near future [4]. Sri Lanka is a small island nation with a population of over 21 million. When the Sri Lankan context is considered, the overall prevalence of diabetes among the population ages 20 to 79 was reported at 11.3% in 2021 [5]. Approximately 25% of the adult population in Sri Lanka is estimated to have metabolic syndrome [6] and the prevalence of hypertension was shown to be 23.7% of the adult population [7]. Dyslipidaemia is also of staggering numbers, with over 75% of Sri Lankan adults having some type of dyslipidaemia [8]. Coronary artery disease, which mainly results from all the chronic diseases listed above, is the leading cause of mortality in Sri Lanka [9]. These statistics show that non-communicable diseases are at an epidemic proportion in the country.

The management of chronic diseases is multi-faceted, and medication therapy has been linked with better control of these diseases and their complications and with improved overall health [10]. The multidimensional adherence model defined by the World Health Organization (WHO) states that socioeconomic factors play an important part in medication adherence [11]. Non-adherence to prescribed treatment has been linked with a significant increase in all-cause hospitalization and all-cause mortality in patients with type 2 diabetes [12]. Medication non-adherence has been linked to more than 100,000 preventable deaths per year in the United States alone [13]. When the availability of medicines decreases due to various reasons, adherence is disrupted, leading to many adverse health outcomes. In India, the unavailability of medicines at primary health centres and the inefficiency of drug procurement at higher levels have been shown to drive people into poverty just from out-of-pocket expenses for the treatment of non-communicable diseases [14]. Access to basic medicines is limited in many regions in Africa as well, mainly due to economical restrictions and poor government policies. Relying on the importation of medicines and raw materials, which gets hindered by foreign exchange scarcity, has contributed to the problem [15]. It can be postulated that similar circumstances created in Sri Lanka by the economic crisis have led to similar disruptions in compliance with medication therapy.

Access to effective and safe medicines has been named a basic human right by WHO [16]. As Sri Lanka is facing the worst economic crisis in its history, it is important to evaluate how the health aspects of the citizens have been affected so that the available resources can be prioritized upon need. We aim to assess the adherence to medication among Sri Lankans during the economic crisis and the underlying reasons for non-adherence, information which may prove useful in policymaking to minimize dire complications arising from decreased medication use in these unprecedented times.

## 2 Methods

A national-level online survey was carried out among Sri Lanka citizens (≥18 years) using Google Forms from July to August 2022. The survey was shared through social media, such as Facebook, Instagram, Twitter, and WhatsApp. The survey was conducted in accordance with the guidelines outlined in the Helsinki Declaration [17]. The ethical approval was obtained from the Ethics Review Committee, Nawaloka Hospitals Research and Education Foundation, Colombo, Sri Lanka. The participants were recruited voluntarily, and informed consent was obtained prior to filling out the survey questionnaire.

## 3 Questionnaire

The desired data was gathered using a pre-designed Google form questionnaire that was made available in all three of the official languages, English, Sinhala, and Tamil. The questionnaire consisted of two sections: the first section inquired about the respondent's sociodemographic characteristics and the second section was related to questions about medication use. The reliability of the questionnaire was tested through piloting, prior to survey administration. The questionnaire is provided in Appendix A.

The section on sociodemographic information included questions such as gender, age, district (25 districts of Sri Lanka), residential area (grouped as inner city, suburban, rural), ethnicity (Sinhalese, Sri Lankan Tamil, Indian Tamil, Moors, Others), educational status (secondary education or below, tertiary education, degree/ diploma or above), number of family members, children (having children, no children), employment status (employed permanently, employed temporarily, unemployed, retired and full-time student), monthly income (no income, <25,000 LKR, <50,000 LKR, 50,000–100,000 LKR, 100,000–200,000 LKR, >200,000 LKR) and main source of monthly income. To minimize the disclosure of personal information, only the year of birth was requested. The participants' ages were re-divided into the following three groups: 18–30 years; (ii) 31–40 years; and (iii) ≥41 years. The living districts were regrouped as “Colombo” and “other districts” during the analysis. Also, the income groups of 25,000 LKR and 50,000 LKR were pooled into one category. Additionally, the primary income sources were regrouped as “employment in the government or cooperative sector”, “jobs in the private sector,” and “other.”

In the section on medication, the respondents were asked about the use of medication, disease conditions, changes in the use of medication, and reasons for any changes. The same inquiries were repeated if respondents had any other family members or children who regularly took medicine. The participants were required to respond “Yes” or “No” to the question about taking medicine. If they were taking regular medicine, they had to select their disease condition from a list of given diseases (diabetes, hypertension, dyslipidemia, cancer, asthma, arthritis, heart diseases, chronic kidney disease, thyroid-related diseases, neurological diseases, psychiatric diseases, and other diseases). The following five options were offered to respondents when asked about the main changes in their medication intake: (i) no change, (ii) reduced the type of medication intake, (iii) reduced the frequency of intake, (iv) changed the brand of medicine, and (v) have stopped taking medicines completely. The respondents selected one of the six options when asked to list the primary causes of the change in medication: (i) high cost of medicines, (ii) lack of money to buy medicines after purchasing other essential items, (iii) unavailability of medicines in the government/private sector, (iv) unavailability of the preferred brand of medicines, (v) difficulty attending hospital or private clinics, and (vi) have lost interest in my own health due to the current situation in the country.

### 3.1 Statistical analysis

All the variables were analyzed quantitatively and were expressed as a percentage (%) and numbers (n). Descriptive statistics were employed to explore the demographic parameters of the study sample. The general distribution of all the demographic variables was assessed using frequency and percentages for categorical variables and means and standard deviations for continuous variables. Moreover, univariate, and multivariate logistic regression analyses were performed to find the sociodemographic factors that influenced the change in use of medication. The results of logistic regression analyses were expressed as odds ratio (OR) and 95% confidence intervals (95% CI). Statistical significance was accepted as *P* < 0.05. All analyses were carried out with the IBM SPSS statistics version. 23.0 (IBM, Chicago, IL, USA).

## 4 Results

**Fig. 1:**
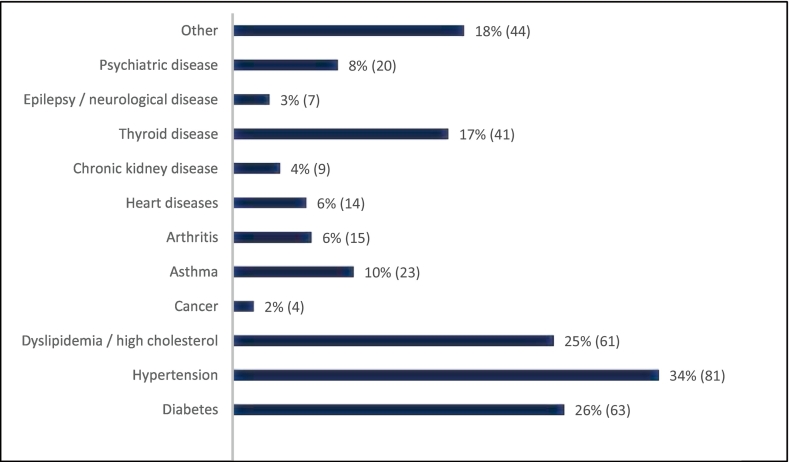
No. of respondents with diseases.

A total of 1214 respondents were included in the analysis after removing incomplete and duplicate records. The participants' sociodemographic characteristics are shown in Table 1. The mean (±SD) age of the participants was 35.12 years (±9.63) and the majority of participants were female (60.0%). Most of the participants were Sinhalese in ethnicity (93%) and represented the Colombo district (42.7%). Compared to 34% of respondents from suburbs and 27.4% from inner cities, 38.6% of respondents were from rural areas. The average family size was 4.11 (±1.29) with more than half of the respondents having 3 to 4 members in their families. However, 63.8% reported that they do not have children. The majority of the participants (84.1%) were having at least a professional degree and were employed either permanently (67.3%) or temporarily (10.9%). Around 84% of respondents said they had a monthly income, and of those who did, 52.8% had monthly incomes of less than 100,000 LKR, and 47.2% had monthly earnings of 100,000 LKR or more. Government or private sector employment accounted for most respondents' primary source of income. Out of the survey participants, nearly 20% were taking regular medication for various chronic diseases. The most common illnesses among respondents who took medication were hypertension (34%), diabetes (26%), and dyslipidemia (25%) (Fig. 1).

**Table 1:** Socio-demographic and socio-economic characteristics of the survey respondents.

Variables	Overall
	n (%)
Age	
18–30 years	456 (37.6)
31–40 years	399 (32.9)
≥41 years	293 (24.1)
Age not reported	66 (5.4)
Gender	
Male	486 (40.0)
Female	728 (60.0)
District	
Colombo	518 (42.7)
Others	696 (57.3)
Area of residence	
Inner-city	333 (27.4)
Suburban	413 (34.0)
Rural	468 (38.6)
Education	
Secondary education (up to O/L or below)	22 (1.8)
Tertiary education (up to A/L	171 (14.1)
Degree or above	1021 (84.1)
Employment status	
Employed permanently	817 (67.3)
Employed temporarily	132 (10.9)
Unemployed	130 (10.7)
Retired	31 (2.6)
Full-time student	104 (8.6)
Monthly family income (in LKR)	
No income	199 (16.4)
<50,000	273 (22.5)
50,000–100,000	263 (21.7)
100,000–200,000	273 (22.5)
>200,000	206 (17.0)

Table 2 demonstrates the results of univariate and multivariate logistic regression analysis. According to the univariate analysis, respondents' education level and monthly income were associated with changes in the use of medication. When adjusted for all the variables in the multivariate analysis, the respondents living outside the Colombo district showed relatively increased risk for not adhering to medication (OR = 1.425, 95% CI = 1.02–1.99, *P* = 0.038). Similarly, the respondents having a monthly income of less than 100,000 LKR were twice more likely to change their medication patterns (OR = 2.278, 95% CI = 1.37–3.78, *P* = 0.001) in comparison to the participants who earned more than 100,000 LKR per month.

**Table 2:** Odds Ratios (OR) for the likelihood of change in use of medication by socio-demographic variables.

Variables	Change in use of medication			
	Univariate analysis		Multivariate analysis	
	OR (95% CI)	*p*-value	OR (95% CI)	p-value
Age				
18–30 years	1.065 (0.740–1.533)	0.735	0.774 (0.515–1.163)	0.217
31–40 years	0.950 (0.655–1.377)	0.786	0.891 (0.605–1.311)	0.556
≥ 41 years^⁎^	1		1	
Gender				
Male	1.001 (0.751–1.335)	0.992	1.079 (0.792–1.470)	0.631
Female^⁎^	1		1	
District				
Other	1.284 (0.969–1.702)	0.082	1.425 (1.020–1.992)	0.038
Colombo	1		1	
Area of residence				
Inner-city	1.046 (0.734–1.490)	0.805	1.354 (0.902–2.034)	0.144
Suburban	1.040 (0.751–1.440)	0.812	1.324 (0.910–1.926)	0.142
Rural^⁎^	1		1	
Education				
Up to O/L or below	1.660 (0.552–4.992)	0.367	1.265 (0.409–3.910)	0.683
Up to A/L	1.665 (1.097–2.529)	0.017	1.371 (0.874–2.152)	0.169
Degree or above^⁎^	1		1	
Monthly family income (in LKR)				
No income	2.306 (1.397–3.806)	0.001	2.345 (1.303–4.217)	0.004
< 50,000	2.189 (1.369–3.499)	0.001	2.345 (1.371–4.010)	0.002
50,000–100,000	2.367 (1.483–3.779)	<0.001	2.278 (1.374–3.775)	0.001
100,000–200,000	1.312 (0.816–2.110)	0.263	1.255 (0.757–2.083)	0.379
>200000^⁎^	1		1	

(The reference category is no change in the use of medication. * Reference variable, CI- confidence interval; OR- odds ratio; P- probability value)

**Fig. 2:**
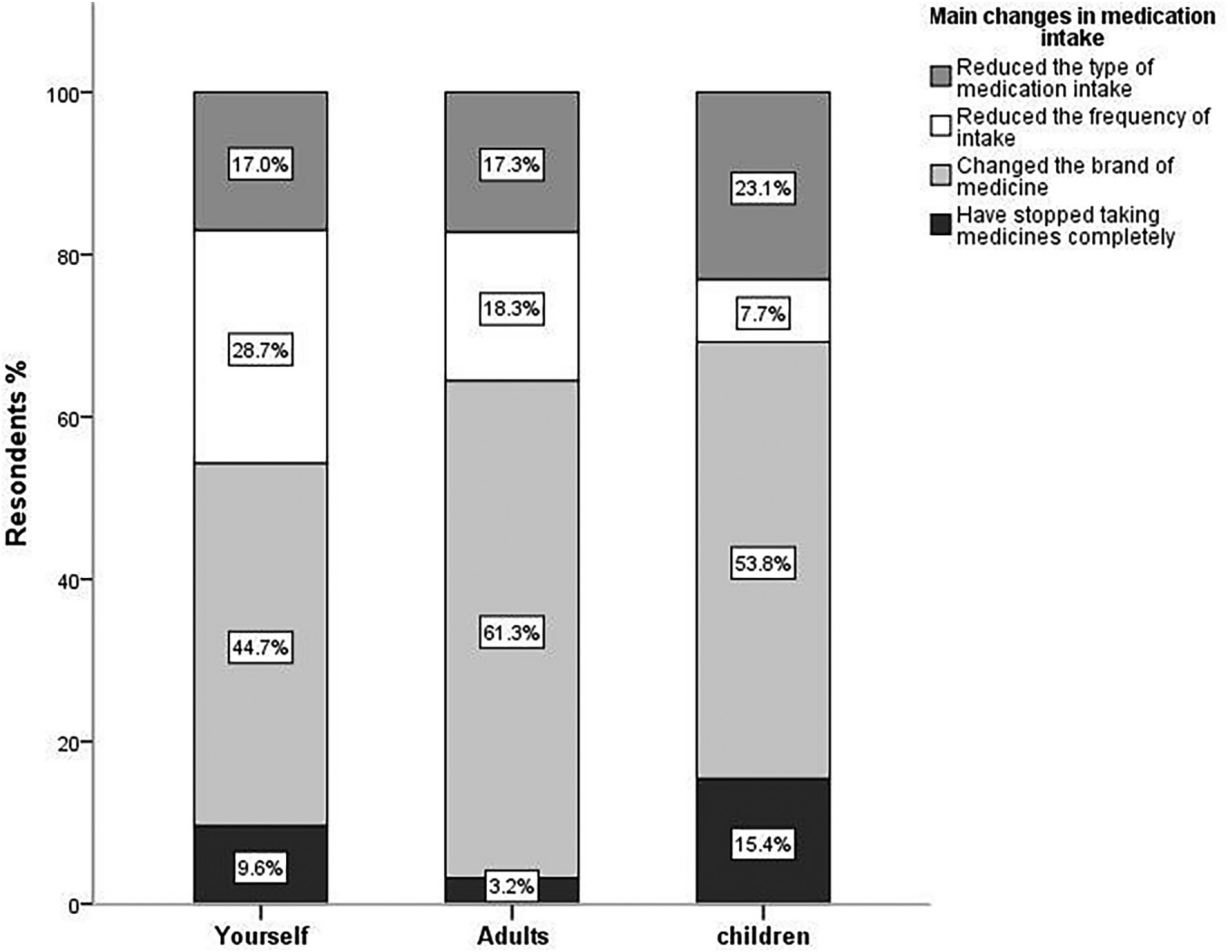
Main changes in medication intake.

As per the survey results, 19.9% of the respondents were taking regular medication. Additionally, 57.7% and 2.9% of participants self-reported having additional adults and children in their families who regularly took medication. Out of the respondents and their family members who used regular medication, 39%, 40.6%, and 37.1% reported that the economic crisis has, respectively, impacted the medication intake in themselves, other adults, and children in their families. Fig. 2 shows the main changes in medication intake during the economic crisis period as compared to the earlier period. The participants identified the primary medication change for themselves (44.7%), other adults (61.3%), and children (53.8%) in families as switching the medication's brand. Reduced medication intake frequency was the second most significant medication change among responders (28.7%) and family members (18.3%). However, in children the second commonest change was the reduction in the type of medication taken (23.1%).

The high cost of medicine was the greatest barrier to medication adherence for participants as well as other adults in their families. Out of the participants taking medication and with other family members taking medication, 47.9% and 41.9% of the respondents reported that the prices were out of reach for both themselves and other adults in their families. Out of the participants who had children taking medication, 46.2% reported the unavailability of medicines in the government/private sector as the primary reason for noncompliance in children, while another 38.5% of respondents stated the high cost of medications as the main reason (Supplementary file 1).

## 5 Discussion

This is the first survey on the changes in medication adherence among Sri Lankans during the current economic crisis. Our study population mainly comprised educated individuals residing in inner city areas and the suburban regions of the country, and more than 70% of the respondents were employed either permanently or temporarily. The predilection towards more urban and more educated participants probably stems from the mode of data collection; namely, social media. It was concerning to observe that more than one-third of such a population has experienced a reduction in their medication adherence due to the adverse financial situation in Sri Lanka. A similar picture is seen in Lebanon, where the economic and socio-political collapse since 2019 has paralyzed their once-sophisticated healthcare system. Subsidies on many medications have been lifted and subsequently, the prices have steeply risen, significantly increasing the number of hospital admissions due to the worsening of chronic conditions as people were unable to afford antidiabetic drugs, inhalers, diuretics, and other medication [18]. The economic crisis around 2014 caused many patients to cut down on their regular medications in Greece as well [19]. Economic constraints and poor government policies have contributed to poor control of chronic diseases such as hypertension in Sub-Saharan Africa [20].

It is identified that South Asian individuals are at an increased risk of diabetes and cardiovascular disease at lower body mass index (BMI) values and younger ages than other ethnic groups [[21], [22], [23], [24]]. The increased risk appears to persist even when South Asians migrate to Western societies [25,26]. Several factors are believed to be underlying this phenomenon. These factors include increased intake of carbohydrates and fat, lower intake of fruits and vegetables, sedentary lifestyle, positive family history, and adverse perinatal environment [27]. Burdened with an epidemic of metabolic syndrome, South Asians, Sri Lankans included, need to thoroughly follow the management strategies, which largely involve long-term pharmacotherapy. Poor compliance with prescribed drugs leads to dire outcomes. Out of all medication-related admissions in the United States, up to two-thirds are caused by non-adherence to medication [28]. Antihypertensive, anti-diabetes, and lipid-lowering therapies are known to significantly lower the risk of future ischaemic events [[29], [30], [31]]. For every 20 mmHg increase in systolic blood pressure and every 10 mmHg increase in diastolic blood pressure, the risk of ischaemic heart disease and stroke doubles [32]. Non-adherence to antiplatelets is also associated with a higher risk of myocardial infarction, stroke, and death [[33], [34], [35]] while the risk of stent thrombosis rises in stent recipients who discontinue clopidogrel prematurely [35,36]. It is apparent that poor compliance with medication therapy for chronic illnesses results in multiple preventable complications and even death, which then adversely affect the patients' families and the country.

Our survey demonstrates that patients living outside the Colombo area and those with lower monthly incomes were more likely to report medication non-adherence. The current economic crisis in the country comes with an unprecedented rise in inflation and reduced foreign exchange reserves, severely limiting the supply of commodities such as fuel, cooking gas, and electricity. Food prices are at an all-time high. It is only logical that people cover their basic day-to-day requirements of food, transportation, water, and electricity with the limited funds available, and cut down on their medication expenses, as the deadly effects of medication non-compliance tend to come at a slower rate compared to the immediate effects of hunger, power cuts, etc.

Studies have shown that the prevalence of metabolic syndrome rises with increasing age [6]. Although the average age of the respondents in our study was around 35 years, 60% of other adults living in their households were on medication for long-term illnesses. The most frequent change in medication use during the economic crisis was switching brands of medicines. The import restrictions imposed to battle the foreign currency deficit may have hindered the supply of previously well-circulated and well-known brands. Patients might have been forced to downgrade from higher-quality medicines to lower-quality ones due to limited availability. Branded medicines and their generic copies have been shown to differ in their bioavailability and efficacy [37]. For example, generic bisphosphonates were associated with lower increases in bone mineral density compared to branded medicines in the management of osteoporosis [38]. In patients on antiepileptic therapy, switching from a branded product to a generic medicine resulted in breakthrough seizures and more adverse outcomes [39,40]. Although the active pharmaceutical ingredient does not differ between brands of the same medicine, other chemicals within the formulation, known as excipients, differ and may cause harm to the patient. Allergic reactions have been reported to croscarmellose sodium (an excipient) in patients switched to a generic form of frusemide. They had been taking a branded product of frusemide without issue previously [41]. This demonstrates the potential dire outcomes switching between brands might bring patients on treatment for chronic illnesses.

Limitations of our study include the inability to measure the effects of medication non-compliance such as poor glycaemic and blood pressure control as we collected data online. The use of online platforms to collect data may have prevented reaching parts of the society most severely affected by the current economic hardships. However, even the more affluent citizens of the country who participated in the survey were significantly affected regarding pharmacotherapy of chronic illnesses during the economic crisis, indicating that the problem is likely worse among poorer people. While switching brands was the most common change in medication use, the unavailability of medicines in the government and private sectors and the high cost were quoted as the main reasons for decreased compliance. Authorities should take prompt action to improve the availability of good-quality medicines in sufficient amounts or the adverse effects of reduced compliance will soon manifest, further worsening health costs and reducing valuable manpower. Local production of essential medicines with ensured quality, regulation of medical prescription and taking measures to avoid dispensing unnecessary drugs, auditing the actual requirements of individual drugs, and utilizing funds to purchase medicines to match their demand and minimizing corruption involved in medication purchasing procedures are some of the measures that can be taken to improve the situation. Unless prompt corrective measures are taken, we will have to face the terrible consequences of poorly managed chronic illnesses in the near future as a country.

## 6 Conclusion

The current economic crisis in Sri Lanka has caused significant changes in medication adherence among patients with chronic illnesses. Even though the respondents to this study were well-educated and wealthy inhabitants of the country, a substantial proportion claimed that the economic crisis had negatively affected the medication intake in themselves, other adults, and children in their families. Respondents reporting medication non-adherence were more likely to reside outside of the Colombo region and have lower monthly salaries. Quick interventions are required to minimize the adverse outcomes of poor control of such diseases.

## Authors' contribution

PS and RJ conceived and designed the online survey questionnaire; distributed the questionnaire; PS analyzed and interpreted the data; PS and WK drafted the manuscript; RJ revised the manuscript. All authors read and approved the final manuscript.

## Funding

No funding was received for conducting this study.

## Ethical approval

The ethical approval was obtained from the Ethics Review Committee, Nawaloka Hospitals Research and Education Foundation, Colombo, Sri Lanka. Informed consent was obtained from the participants before filling the survey questionnaire.

## Declaration of Competing Interest

The authors declare no competing financial interests or personal relationships that could have appeared to influence the work reported in this paper.

## Appendix A Supplementary data

Supplementary material 1 Image 1

Appendix A Image 2
