# Supplementary material for: The impact of the Sri Lankan economic crisis on medication adherence: An online cross-sectional survey
Source: Dialogues Health. 2023 May 18;2:100137. doi: 10.1016/j.dialog.2023.100137 (PMC10953977; doi:10.1016/j.dialog.2023.100137)
Supplement: Supplementary file 1 — Supplementary material 1 [file mmc1.docx]

**Supplementary file 1**

| **About yourself** |  |
| --- | --- |
| **Having medication** |  |
| Yes | 241 (19.9) |
| No | 973 (80.1) |
| **Is there any 𝐦𝐚𝐢𝐧 change in your medication intake** |  |
| No change | 147 (61.0) |
| Reduced the type of medication intake | 16 (6.6) |
| Reduced the frequency of intake | 27 (11.2) |
| Changed the brand of medicine | 42 (17.4) |
| Have stopped taking medicines completely | 9 (3.7) |
| **Reasons for change** |  |
| High cost of medicines | 45 (47.9) |
| Lack of money to buy medicines after purchasing other essential items | 14 (14.9) |
| Unavailability of medicines in the government/private sector | 15 (16.0) |
| Unavailability of the preferred brand of medicines | 11 (0.9) |
| Difficulty attending hospital or private clinics | 1 (0.1) |
| Have lost interest in my own health due to the current situation of the country | 8 (8.5) |
| **Adults** |  |
| **Having medication** |  |
| Yes | 700 (57.7) |
| No | 514 (42.3) |
| **Is there any main change in medication intake in adults in your family** |  |
| No change | 416 (59.4) |
| Reduced the type of medication intake | 49 (7.0) |
| Reduced the frequency of intake | 52 (7.4) |
| Changed the brand of medicine | 174 (24.9) |
| Have stopped taking medicines completely | 9 (1.3) |
| **Reasons for change** |  |
| High cost of medicines | 119 (41.9) |
| Lack of money to buy medicines after purchasing other essential items | 27 (9.5) |
| Unavailability of medicines in the government/private sector | 74 (26.1) |
| Unavailability of the preferred brand of medicines | 52 (18.3) |
| Difficulty attending hospital or private clinics | 9 (3.2) |
| Have lost interest in my own health due to the current situation of the country | 3 (1.1) |
| **Children** |  |
| **Having medication** |  |
| Yes | 35 (2.9) |
| No | 1179 (97.1) |
| **Is there any main change in medication intake in children in your family** |  |
| No change | 22 (62.9) |
| Reduced the type of medication intake | 3 (8.6) |
| Reduced the frequency of intake | 1 (2.9) |
| Changed the brand of medicine | 7 (20.0) |
| Have stopped taking medicines completely | 2 (5.7) |
| **Reasons for change** |  |
| High cost of medicines | 5 (38.5) |
| Lack of money to buy medicines after purchasing other essential items | 0 |
| Unavailability of medicines in the government/private sector | 6 (46.2) |
| Unavailability of the preferred brand of medicines | 1 (7.7) |
| Difficulty attending hospital or private clinics | 0 |
| Have lost interest in my own health due to the current situation of the country | 1 (7.7) |
