# Supplementary material for: The impact of the Sri Lankan economic crisis on medication adherence: An online cross-sectional survey
Source: Dialogues Health. 2023 May 18;2:100137. doi: 10.1016/j.dialog.2023.100137 (PMC10953977; doi:10.1016/j.dialog.2023.100137)
Supplement: Supplementary file 2 — Appendix A [file mmc2.pdf]

THE IMPACT OF THE ECONOMIC CRISIS ON LIFESTYLE OF SRI LANKANS

This short survey is designed to capture changes in lifestyle and dietary patterns during the ongoing economic crisis in the country. Your participation and honest responses will be highly appreciated. The questionnaire takes about 10-15 minutes to complete. The information you provide will be kept strictly confidential.

* Required

1. Please tick here for your informed consent for agreeing to participate in this survey. *

Mark only one oval.

- ⬭ I agree
- ⬭ I don't agree

About Yourself

2. 1. What is your gender? *

Mark only one oval.

- ⬭ Male
- ⬭ Female
- ⬭ Other

3. 2. What is your birth date? *

____

Example: January 7, 2019

4. 3. What is your highest educational level? *

Mark only one oval.

- ⬭ Up to grade 5 or below
- ⬭ Secondary education (up to O/L)
- ⬭ Tertiary education (up to A/L)
- ⬭ Degree/ diploma or above

5. 4. What is your district of living? *

Mark only one oval.

- Ampara
- Anuradhapura
- Badulla
- Batticaloa
- Colombo
- Galle
- Gampaha
- Hambantota
- Jaffna
- Kalutara
- Kandy Kegalle
- Kilinochchi
- Kurunegala
- Mannar
- Matale
- Matara
- Monaragala
- Mullaitivu
- Nuwara Eliya
- Polonnaruwa
- Puttalam
- Ratnapura
- Trincomalee
- Vavuniya

6. 5. What is the nature of your living area? *

Mark only one oval.

- Inner-city
- Suburban
- Rural

7. 6. What is your ethnicity? *

Mark only one oval.

- Sinhala
- Sri Lankan Tamil
- Indian Tamil
- Sri Lankan Moors
- Others

8. 7. What is your current employment status? *

Mark only one oval.

- Employed permanently
- Employed temporarily
- Unemployed
- Retired from employment
- Full-time student (unemployed)

9. 8. How many members are there in your family **including yourself**? *

Mark only one oval.

- 1
- 2
- 3
- 4
- 5
- 6
- 7
- 8
- 9
- 10 or more

10. 9. Please indicate the number *

	0	1	2	3	4	5	6	7
Chiilldrren ((age ≤ 10))								
Adollescentts ((11--17))								
Adulltts ((18–65))								
Ellderrlly ((>65))								

11. 10. Do you have a monthly income? *

Mark only one oval.

- Yes *Skip to question 12*
- No *Skip to question 14*

Income Related

12. 11. What is your **main** source of monthly income? *

Mark only one oval.

- Government or cooperative sector jobs
- Private sector jobs
- Agricultural or animal husbandryFishery
- Labor jobs
- Own business
- Other cash income

13. 12. How much is your monthly income (LKR)? *

Mark only one oval.

- Less than 25,000
- 25,000-50,000
- 50,000-100,000
- 100,000-200000
- >200000

Adherence to Medication

44. 40. Do you take medication for any chronic disease or any other disease? *

Mark only one oval.

- ( ) Yes
- ( ) No *Skip to question 48*

Your Medication

- [ ] Diabetes
- [ ] Hypertension
- [ ] Dyslipidemia / high cholesterol
- [ ] Cancer
- [ ] Asthma
- [ ] Arthritis Heart
- [ ] disease
- [ ] Chronic kidney disease
- [ ] Thyroid disease
- [ ] Epilepsy / neurological disease
- [ ] 11. Psychiatric disease
- [ ] 12. Other

46. 42. If you take medicine, is there any **main** change in your medication intake due to the crisis? *

Mark only one oval.

- ( ) No change *Skip to question 48*
- ( ) Reduced the type of medication intake
- ( ) Reduced the frequency of intake
- ( ) Changed the brand of medicine
- ( ) Have stopped taking medicines completely

*

47. 43. If there is a change in your medication intake, it is **mainly** because of the?

Mark only one oval.

- High cost of medicines
- Lack of money to buy medicines after purchasing other essential items
- Unavailability of medicines in the government/private sector Unavailability
- of the preferred brand of medicines
- Difficulty attending hospital or private clinics
- I have lost interest in my own health due to the current situation of the country

Medicines for Adults

48. 44. Are there any other adults in your household who are taking regular medication for any chronic diseases? *

Mark only one oval.

- Yes
- No *Skip to question 51*

49. 45. If yes, is there any **main** change in their medication intake due to the crisis? *

Mark only one oval.

- No change *Skip to question 51*
- Reduced the type of medication intake
- Reduced the frequency of intake
- Changed the brand of medicine
- Have stopped taking medicines completely

*

50. 46. If there is a change in their medication intake, it is **mainly** because of the?

Mark only one oval.

- High cost of medicines
- Lack of money to buy medicines after purchasing other essential items
- Unavailability of medicines in the government/private sector Unavailability
- of the preferred brand of medicines
- Difficulty attending hospital or private clinics
- I have lost interest in my own health due to the current situation of the country

Medicines for Children

51. 47. Are there any children in your household who are taking regular medication for any chronic diseases? *

Mark only one oval.

- Yes
- No *Skip to question 56*

52. 48. If yes, is there any **main** change in their medication intake due to the crisis? *

Mark only one oval.

- No change *Skip to question 56*
- Reduced the type of medication intake
- Reduced the frequency of intake
- Changed the brand of medicine
- Have stopped taking medicines completely

*

50.

Mark only one oval.

- High cost of medicines
- Lack of money to buy medicines after purchasing other
- essential items Unavailability of medicines in the
- government/private sector Unavailability of the preferred
- brand of medicines
- Difficulty attending hospital or private clinics
- I have lost interest in my own health due to the current situation of the country

54. 50. If you or anyone in your family has a change in the medication intake dueto any reason other than those listed above, please mention that reason.

Disease Control

55. 51. Do you think anyone in your family including yourself has lost control of an illness as a result of not taking medication properly because of the economic crisis?

Mark only one oval.

- Yes
- No
- None of my family members take regular medication
